# Study on TCM Syndrome Differentiation of Primary Liver Cancer Based on the Analysis of Latent Structural Model

**DOI:** 10.1155/2015/761565

**Published:** 2015-03-01

**Authors:** Zhan Gu, Xiuzhong Qi, Xiaofeng Zhai, Qingbo Lang, Jianying Lu, Changping Ma, Long Liu, Xiaoqiang Yue

**Affiliations:** ^1^Changhai Hospital of Traditional Chinese Medicine, Second Military Medical University, Shanghai 200433, China; ^2^Anhui Jing'an Hospital of Integrated Traditional Chinese and Western Medicine, Hefei 230031, China

## Abstract

Primary liver cancer (PLC) is one of the most common malignant tumors because of its high incidence and high mortality. Traditional Chinese medicine (TCM) plays an active role in the treatment of PLC. As the most important part in the TCM system, syndrome differentiation based on the clinical manifestations from traditional four diagnostic methods has met great challenges and questions with the lack of statistical validation support. In this study, we provided evidences for TCM syndrome differentiation of PLC using the method of analysis of latent structural model from clinic data, thus providing basis for establishing TCM syndrome criteria. And also we obtain the common syndromes of PLC as well as their typical clinical manifestations, respectively.

## 1. Introduction

Primary liver cancer (PLC) is one of the most common malignant tumors worldwide because of its high incidence and high mortality [1, 2]. It is also one of the most threatening cancers in China, with the highest incidence rate in the world [3]. As one of the most popular complementary and alternative medicine, traditional Chinese medicine (TCM) plays an active role in the prevention and treatment of PLC in China [4]. The advantages of TCM in the treatment of PLC are valued by an increasing number of patients.

PLC is regarded as “abdominal mass” with diverse etiologies, various clinical manifestations, and complicated syndromes in TCM [5]. There are currently no widely accepted guidelines on the classification of patients with PLC into ZHENG types. ZHENG, also known as TCM syndrome, is an integral and essential part of TCM theory [6]. The syndrome based on the clinical manifestations from traditional four diagnostic methods (including inspection, listening, inquiry, and palpation) can be differentiated by a clinical TCM practitioner rather than microlevel laboratory tests or imaging examination. The accurate differentiation of a specific syndrome is the foundation of an effective and individualized treatment strategy, as well as the key to recognizing the disease state [7]. Therefore, syndrome differentiation plays the most important role in the TCM system, and syndrome research has always been hot and difficult spots in TCM basic studies [8]. However, the syndrome research is suffering the question of credibility because of the lack of statistical validation support.

TCM diagnosis consists of two steps. One is called “patient information gathering” and the other is known as “syndrome differentiation.” Subjectivity is one of the issues in both of the two steps and we focus more on the latter. So establishing objective and quantitative standards for syndrome differentiation has become one of the main directions of syndrome research. In recent years, the research in syndrome standardization has made certain progress because of the advantage of the modern science and technology. The analysis of latent structural model (LSM), one of the unsupervised data analysis methods, is put forward specifically for TCM syndrome research [9]. In this study, LSM implements multidimensional hierarchical clustering targeting the manifest variables by introducing latent variables. The manifest variables consist of specific clinical manifestations from traditional four diagnostic methods in patients with PLC which can be directly observed. And the latent variables consist of different levels of combinations of symptoms and signs which can be obtained only through comprehensive analysis, including the basic TCM pathogenesis law and the main syndrome factors. Finally, LSM can mine the potential characteristics of the latent variables adequately by analyzing the connection between latent variables and manifest variables, as well as the connection among latent variables themselves. All of the above indicates that the core TCM syndrome of PLC could be identified by the method of LSM analysis.

## 2. Materials and Methods

### 2.1. Review of LSM

LSM is often used in data-driven medical research, which implements multidimensional hierarchical clustering targeting the manifest variables by introducing latent variables to achieve the goal of mining potential characteristics of the latent variables. It also can reveal and evaluate the connection between the clinical manifestations and the syndrome visually and objectively through the objective data. It has been applied in the study of syndrome differentiation in certain diseases in recent years. For example, based on the TCM inquiry information, Xu established the database of 3021 patients with cardiovascular disease (CVD). Evaluating the TCM inquiry information with LSM, Xu concluded that, in the crowd of patients with CVD, syndromes involved in deficiency syndrome mainly contained Qi deficiency, Yang deficiency, deficiency of Qi and Yin, and body fluid impairment. And syndromes involved in excess syndrome mainly contained Qi-stagnation, phlegm-dampness, blood-stasis, and heat. Xu also suggested the correlation between the syndrome and the clinical manifestations [10, 11].

As one of the data mining techniques, LSM analysis can be used to reveal the laws about symptom occurrence and cooccurrence systematically and obtain multiple classifications reflecting the patients' conditions from different points of view. Therefore, LSM analysis can provide objective basis for syndrome research. LSM is finished by using a new class of probabilistic model which is developed by the Department of Computer Science and Engineering at Hong Kong University of Science and Technology [9]. Referring to the dialectical thinking mode of TCM and implementing hierarchical clustering by a Bayesian network-based latent tree model (also known as hierarchical latent structural model), LSM can evaluate the clinical manifestations from traditional four diagnostic methods synthetically and reveal potential laws to guide the syndrome differentiation [12]. A java implementation of EAST is used to analyze the data and a layered exploration is carried out [13].

### 2.2. Collection of Clinic Data

A total of 559 effective cases of in-patients with PLC, aged from 27 to 78, were collected from Shanghai Changhai Hospital of Traditional Chinese Medicine (from January 1, 2005, to September 30, 2006), including 482 male cases and 77 female cases. Diagnosis and staging standards of PLC referred to “the standard of clinical diagnosis and staging of primary liver cancer” (Chinese Society of Liver Cancer, 2001) [14]. Clinical stage distribution of all selected patients is as follows: 66 cases of Ia stage and 72 cases of Ib stage, 92 cases of IIa stage and 171 cases of IIb stage, and 139 cases of IIIa stage and 19 cases of IIIb stage. All the patients had signed informed consents voluntarily.

The clinical manifestations (symptoms and signs) of in-patients with PLC from traditional four diagnostic methods were collected by the “questionnaire about TCM syndrome of liver cancer.” This questionnaire was designed by Shanghai Changhai Hospital of Traditional Chinese Medicine based on literature analysis, experts' discussion, and clinical verification in our earlier study [15]. Various measures were taken to ensure data quality. For example, the questionnaires were completed by TCM practitioners after unified staff training, and the judgments of tongue appearance and pulse pattern were obtained by two senior doctors together who had more than ten years of TCM experiences. In the process of information collection, TCM syndrome is not given in order to minimize the selective and measurement bias.

### 2.3. Entry of Data

The collected clinical manifestations of in-patients with PLC were marked “1” or “0” respectively. Patients marked “1” were accompanied with corresponding symptoms or signs, while patients marked “0” were not. Then the marked results were input into an access database designed for the patients with PLC, which was developed by Shanghai Changhai Hospital of Traditional Chinese Medicine. In order to ensure the accuracy of the data, we adopted dual data entry, contrastive check, and sampling inspection (sampling rate was not less than 30%). By means of frequency analysis, literature review, and experts' consultation, 57 common symptoms and signs were finally entered in the follow-up analysis including hypochondriac pain, spontaneous sweating, fatigue, lumbar genu aching and limp, insomnia, night urination, bad breath, shoulder and back pain, yellow urine, hiccup, edema in feet and legs, jaundice, heat in palms and soles, thirst, bitter taste, dry mouth and throat, heavy head and body, depression, abdominal distension, stomach bloating, stomachache, chest pain, chest distress, dizziness, chills, hydrothorax, hypochondrium block, oliguria, nausea and vomiting, anorexia, loose stool, tinnitus, night sweating, dim complexion, pale complexion, sallow complexion, greasy tongue coating, yellow tongue coating, white tongue coating, unsmooth pulse, dry stool, sublingual vein varicose, petechiae tongue, plump tongue, purplish tongue, red tongue, slippery pulse, taut pulse, thready pulse, weak pulse, liver palms and spider nevus, cyanotic lips and nails, rapid pulse, ascites, hectic fever, fever, and pale lips and nails.

### 2.4. Statistics of Data

**Figure 1:**
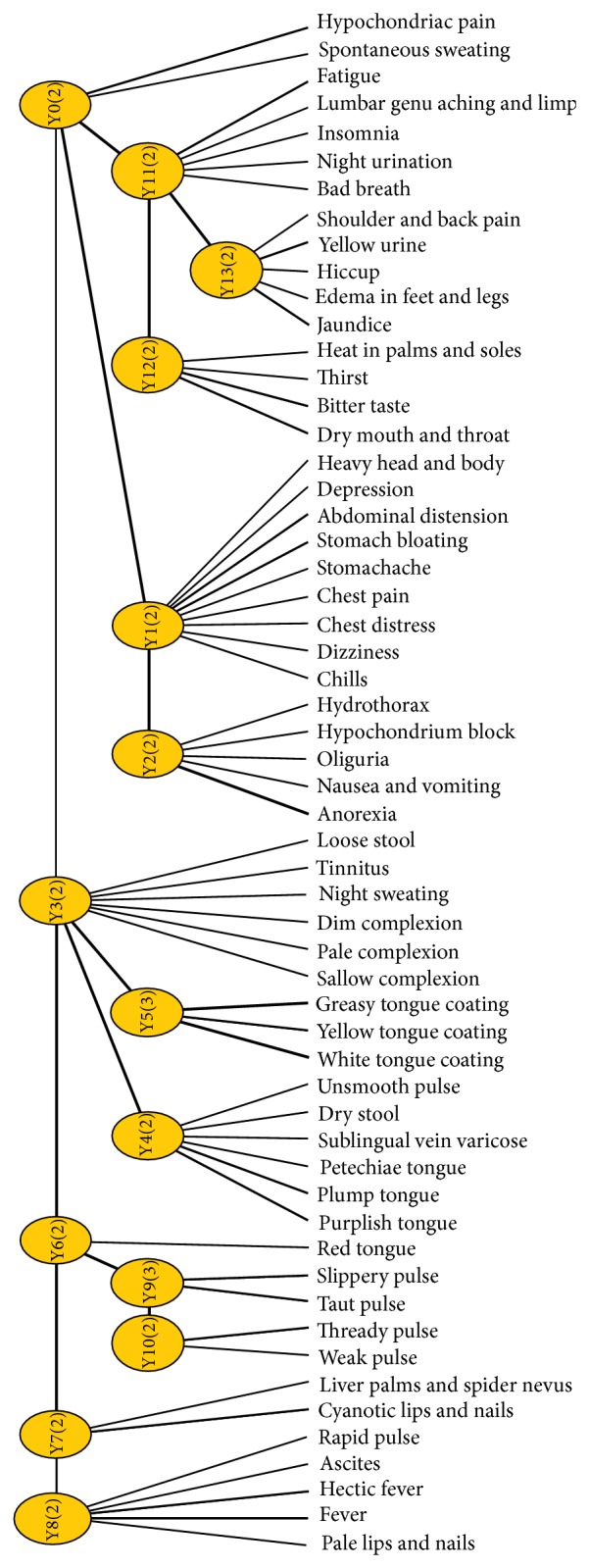
The LSM of 57 inquiry clinical manifestations of 559 cases.

The Lantern 3.1.2 software [16], developed by the Department of Computer Science and Engineering at Hong Kong University of Science and Technology, was used to analyze the data under its EAST algorithm. First, we established the LSM (Figure 1) to form a tree structure to show the relationship between the manifest variables and the latent variables, by which the pathogenesis of each latent variable was given based on the analysis of correspondingmanifest variables according to TCM theory. Second, the syndrome factors, basic units to form the syndrome [17], were extracted from the pathogenesis of each latent variable. Then the latent variables containing the same syndrome factors were analyzed by joint clustering and the information curves were drawn to screen the corresponding manifest variables belonging to the syndrome factors.

## 3. Results

### 3.1. The Analysis of Pathogenesis Law of Each Latent Variable

As shown in the tree structure, there are 14 latent variables named from Y0 to Y13. Each latent variable in the model represents one dimension along which we clustered the data set, and different latent variables represent different ways to cluster the data set [18]. And the correlation strength between variables is shown as the line width. For example, the dependence of fever and hectic fever on latent variable Y8 is stronger than that of rapid pulse, ascites, and pale lips and nails on latent variable Y8. Also, we can accurately obtain the significant manifest variables involved in each latent variable by analyzing their information curves. Then we can understand the underlying law characteristics of the pathogenesis reflected by the significant manifest variables.

**Figure 2:**
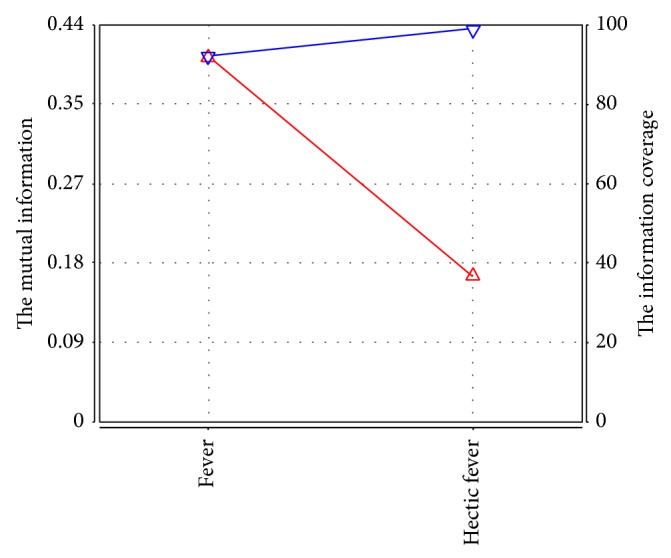
The information curve of latent variable Y8.

In a standard information curve, the curve underneath is the pair wise mutual information curve and the curve above is the cumulate mutual information curve [9, 18]. The abscissa records the manifest variables, which are arranged in the order of the degree of the pair wise mutual information with the corresponding latent variable, namely, in the order of the important degree for the latent variable. The left of ordinate records the mutual information and the right of ordinate records the information coverage. The extracted information can reflect the basic situation of the latent variable when the information coverage reaches 95% [19]. As shown in Figure 2, the clinical manifestations arranged in the order of the important degree for Y8 are successively fever and hectic fever. While the two symptoms are enough to reflect the basic situation of latent variable Y8.

Based on the information curve and TCM theory, we conclude that symptom involved in latent variable Y0 mainly contains hypochondriac pain, which is related to Qi stagnation and blood stasis. Similarly, Y1 successively contains stomach bloating, chills, chest distress, stomachache, dizziness, and abdominal distension, mainly related to Qi stagnation. Y2 contains anorexia and hypochondrium block, related to deficiency of healthy-Qi with excess pathogenic factors. Y3 contains dim complexion, night sweating, loose stool, tinnitus, and sallow complexion, related to deficiency of spleen and kidney. Y4 contains purplish tongue, plump tongue, sublingual vein varicose, and petechiae tongue, related to blood stasis based on the pathological classification of tongue appearance. Y5 contains greasy tongue coating, yellow tongue coating, and white tongue coating, related to endogenous dampness (cold or hot) based on the pathological classification of tongue appearance. Y6 only contains red tongue, suggesting that it may be related to heat. Y7 contains cyanotic lips and nails, liver palms, and spider nevus, related to blood stasis. Y8 contains fever and hectic fever, related to heat. Y9 and Y10 are both pulse manifestations, related to excess and deficiency, respectively. Y11 contains fatigue, lumbar genu aching and limp, insomnia, and night urination, related to deficiency of liver and kidney. Y12 contains dry mouth and throat, bitter taste, and heat in palms and soles, related to Yin deficiency generating intrinsic heat. Y13 contains yellow urine, hiccup, jaundice, and edema in feet and legs, related to dampness-heat of liver and gallbladder.

### 3.2. The Main Syndrome Factors of Each Latent Variable Interpreted by TCM Theory

Syndrome factor is the basic composition unit of the syndrome in TCM and a syndrome factor is also a single syndrome [17]. For example, Qi-stagnation and blood-stasis syndrome contain two syndrome factors (including Qi-stagnation syndrome and blood-stasis syndrome). Combining with the above understanding of the pathogenesis law interpreted by the latent variables, we take the syndrome factor as the breakthrough point, summarizing the main syndrome factors interpreted by the latent variables as follows.

Latent variable Y0 mainly contains Qi-stagnation and blood-stasis; Y1 contains Qi-stagnation; Y2 contains deficiency of healthy-Qi (including Qi deficiency and deficiency of spleen) and blood-stasis; Y3 contains deficiency of healthy-Qi (including Yang deficiency, blood deficiency, deficiency of spleen, and deficiency of kidney); Y4 contains blood-stasis; Y5 contains dampness; Y6 may be related to heat; Y7 contains blood-stasis; Y8 is related to heat; Y9 reflects syndrome factor mainly related to excess; Y10 reflects syndrome factor mainly related to deficiency; Y11 contains deficiency of healthy-Qi (including Qi deficiency, deficiency of liver, and deficiency of kidney); Y12 contains Yin deficiency (belonging to deficiency of healthy-Qi) and heat; Y13 contains dampness and heat.

### 3.3. Joint Clustering of Latent Variables with Same Syndrome Factor

Joint clustering is the subsequent handling-procedure of latent structural data modeling [20]. The results showed that multiple latent variables are sometimes related to one type of syndrome or syndrome factor at the same time and reflect different aspects. Therefore we need to consider the main clinical manifestations from traditional four diagnostic methods involved in these latent variables, comprehensively, then implement joint clustering targeting these latent variables, and obtain some sort of joint clustering model (JCM). The qualitative relationship between the syndrome or syndrome factor and the manifestations by the information curve can be obtained.

Using the Lantern 3.1.2 software, five types of syndrome factors (including Qi-stagnation, dampness, blood-stasis, heat, and deficiency of healthy-Qi) were selected, which have important influence on the pathogenesis law of PLC according to TCM theory and the above analysis. Then the joint clustering of the corresponding latent variables were made and their relevant information curves were obtained.

**Figure 3:**
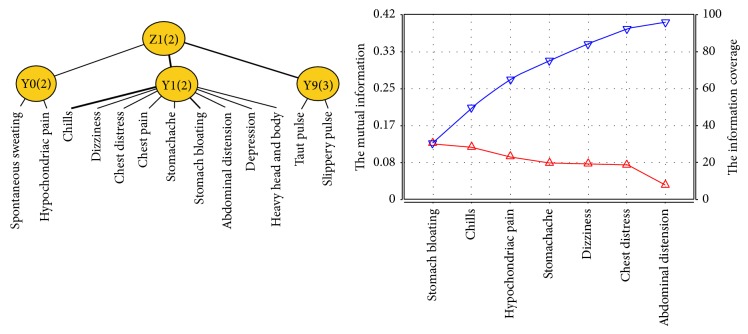
The JCM and information curve of latent variable Z1.

Qi-stagnation syndrome factor is related to latent variables Y0, Y1, and Y9. Z1 was named as a new latent variable and connected to latent variables Y0, Y1, and Y9. After joint clustering we got the JCM and information curve (Figure 3) of Z1. Symptoms and signs involved in Z1 successively contain stomach bloating, chills, hypochondriac pain, stomachache, dizziness, chest distress, and abdominal distension. According to the professional knowledge, chills and dizziness were excluded because of the weak relationship between the two manifestations and Qi-stagnation syndrome. Therefore, the typical clinical manifestations of Qi-stagnation syndrome of patients with PLC include hypochondriac pain, stomach bloating and pain, abdominal distension, and chest distress.

**Figure 4:**
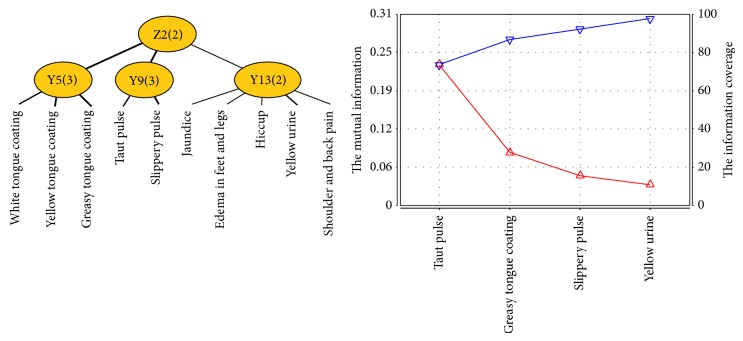
The JCM and information curve of latent variable Z2.

Dampness syndrome factor is related to latent variables Y5, Y9, and Y13. Z2 was named as a new latent variable and connected to latent variables Y5, Y9, and Y13. After joint clustering, we got the JCM and information curve (Figure 4) of Z2. Symptoms and signs involved in Z2 successively contain taut pulse, greasy tongue coating, slippery pulse, and yellow urine. Therefore, the typical clinical manifestations of dampness syndrome of patients with PLC include yellow urine, greasy tongue coating, taut, and slippery pulse.

**Figure 5:**
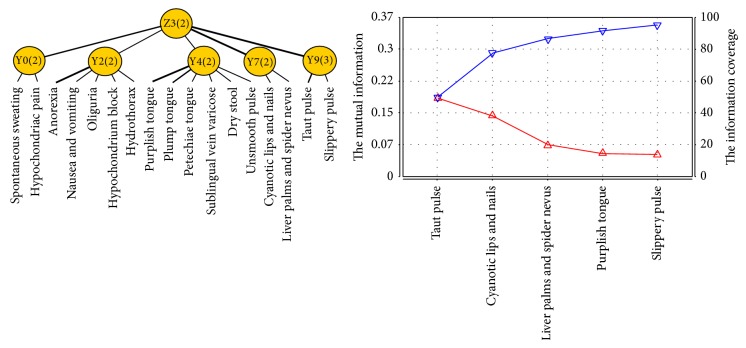
The JCM and information curve of latent variable Z3.

Blood-stasis syndrome factor is related to latent variables Y0, Y2, Y4, Y7, and Y9. Z3 was named as a new latent variable and connected to latent variables Y0, Y2, Y4, Y7, and Y9. After joint clustering, we got the JCM and information curve (Figure 5) of Z3. Symptoms and signs involved in Z3 successively contain taut pulse, cyanotic lips and nails, liver palms and spider nevus, purplish tongue, slippery pulse, and plump tongue. According to the professional knowledge, slippery pulse and plump tongue are excluded because of the weak relationship between the two manifestations and blood-stasis syndrome. Therefore, the typical clinical manifestations of blood-stasis syndrome of patients with PLC include cyanotic lips and nails, liver palms and spider nevus, purplish tongue, and taut pulse.

**Figure 6:**
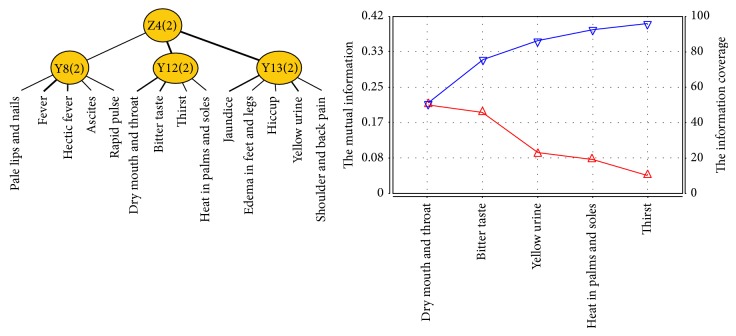
The JCM and information curve of latent variable Z4.

Heat syndrome factor is related to latent variables Y8, Y12, and Y13. Z4 was named as a new variable and connected to latent variables Y8, Y12, and Y13. After joint clustering, we got the JCM and information curve (Figure 6) of Z4. Symptoms and signs involved in Z4 successively contain dry mouth and throat, bitter taste, yellow urine, heat in palms and soles, and thirst. Therefore, the typical clinical manifestations of heat syndrome of patients with PLC include dry mouth and throat, thirst, bitter taste, heat in palms and soles, and yellow urine.

**Figure 7:**
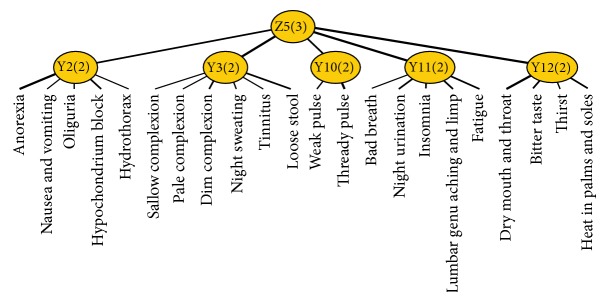
The JCM of latent variable Z5.

Deficiency of healthy-Qi is a summarization of Qi deficiency, blood deficiency, Yin deficiency, Yang deficiency, and deficiency of organs. Deficiency of healthy-Qi syndrome factor is related to latent variables Y2, Y3, Y10, Y11, and Y12. Z5 was named as a new variable and connected to latent variables Y2, Y3, Y10, Y11, and Y12. After joint clustering, the JCM (Figure 7) shows three different conditions in this new latent variable, which means there are two subclassifications in it. According to the professional knowledge, deficiency of healthy-Qi syndrome factor can be divided into two subsyndromes: Yang deficiency of spleen and kidney syndrome (related to latent variables Y2, Y3, and Y10) and Yin deficiency of liver and kidney syndrome (related to latent variables Y10, Y11, and Y12). We name Z5a and Z5b as new variables for the two syndromes and finish joint clustering, respectively.

**Figure 8:**
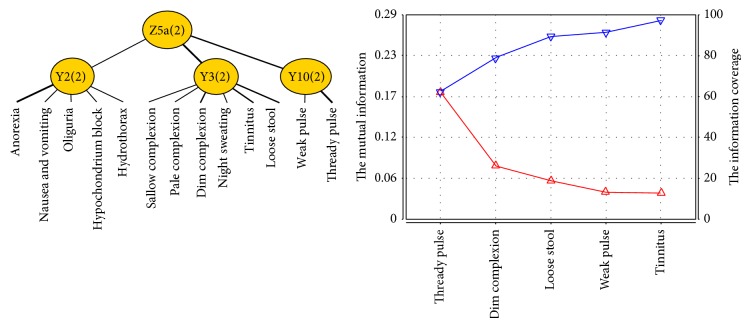
The JCM and information curve of latent variable Z5a.

From the JCM and information curve (Figure 8) of Z5a, we concluded that symptoms and signs involved in Z5a successively contain thready pulse, dim complexion, loose stool, weak pulse, and tinnitus. Therefore, the typical clinical manifestations of Yang deficiency of spleen and kidney syndrome of patients with PLC include dim complexion, tinnitus, loose stool, and thready and weak pulse.

**Figure 9:**
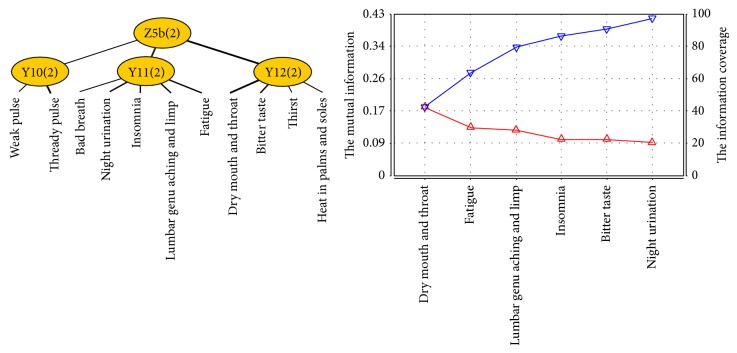
The JCM and information curve of latent variable Z5b.

From the JCM and information curve (Figure 9) of Z5b, we concluded that symptoms and signs involved in Z5b successively contain dry mouth and throat, fatigue, lumbar genu aching and limp, bitter taste, insomnia, and night urination. According to the professional knowledge, bitter taste is excluded because of the weak relationship with Yin deficiency of liver and kidney syndrome. Therefore, the typical clinical manifestations of Yin deficiency of liver and kidney syndrome of patients with PLC include dry mouth and throat, fatigue, lumbar genu aching and limp, insomnia, and night urination.

In conclusion, based on the LSM analysis, the study finally obtained six types of common syndromes of patients with PLC, including Qi-stagnation syndrome, dampness syndrome, blood-stasis syndrome, heat syndrome, Yang deficiency of spleen and kidney syndrome, Yin deficiency of liver and kidney syndrome, and their typical clinical manifestations.

## 4. Discussion

Syndrome differentiation is the basis of clinical treatment in TCM. However, the science of the syndrome has always been questioned for its complicated characteristics of “multidimensional and multistage” and its differentiation depending more on the doctors' subjective judgments. In recent years, the syndrome has become a bottleneck restricting the development of the modernization of TCM.

The method of LSM analysis can provide a basis and prompt for TCM syndrome differentiation, and studies have demonstrated comprehensive cluster analysis and class subdivision application can further clarify the relationship between the latent variables and the manifest variables, thus providing basis for establishing TCM syndrome criteria.

In this study, we collected a total of 559 effective cases of in-patients with PLC, obtained the objective clinical manifestations of them from traditional four diagnostic methods, and used the LSM analysis to explore the syndrome law of PLC. First, we clustered the manifest variables into 14 latent variables. According to the information curves, we could accurately obtain the significant manifest variables involved in each latent variable. Then the pathogenesis of each latent variable was given based on the analysis of correspondingmanifest variables by TCM theory. Second, the main syndrome or syndrome factors of each latent variable were interpreted by TCM theory including Qi-stagnation syndrome, dampness syndrome, blood-stasis syndrome, heat syndrome, and deficiency of healthy-Qi syndrome (including Qi deficiency, blood deficiency, Yin deficiency, Yang deficiency, deficiency of spleen, deficiency of liver, and deficiency of kidney). Then we made joint clustering for the latent variables with same syndrome factors. Finally we preliminarily obtained six types of common syndromes of PLC (Qi-stagnation syndrome, dampness syndrome, blood-stasis syndrome, heat syndrome, Yang deficiency of spleen and kidney syndrome, and Yin deficiency of liver and kidney syndrome) and their typical clinical manifestations.

Although this kind of data mining technology has shown its advantages in the study of syndrome differentiation, it still has some deficiencies at present. Despite the limitations, the present study, for the first time, came to the preliminary conclusion of syndrome differentiation of PLC based on the objective data and mathematical analysis. As establishing objective and quantitative standards for syndrome differentiation has become one of the main directions of TCM syndrome research, we believe that our study will provide a new method for establishing TCM syndrome criteria and should be used for future reliability and validity studies.

## 5. Conclusion

Qi-stagnation, dampness, blood-stasis, heat, Yang deficiency of spleen and kidney, and Yin deficiency of liver and kidney are the common syndromes of PLC and their typical clinical manifestations are also obtained, respectively. Qi-stagnation syndrome includes hypochondriac pain, stomach bloating and pain, abdominal distension, and chest distress. Dampness syndrome includes yellow urine, greasy tongue coating, taut, and slippery pulse. Blood-stasis syndrome includes cyanotic lips and nails, liver palms and spider nevus, purplish tongue, and taut pulse. Heat syndrome includes dry mouth and throat, thirst, bitter taste, heat in palms and soles, and yellow urine. Yang deficiency of spleen and kidney syndrome includes dim complexion, tinnitus, loose stool, and thready and weak pulse. Yin deficiency of liver and kidney syndrome includes dry mouth and throat, fatigue, lumbar genu aching and limp, insomnia, and night urination.

## References

[1] Srivatanakul P., Sriplung H., Deerasamee S. (2004). Epidemiology of liver cancer: an overview. *Asian Pacific Journal of Cancer Prevention*.

[2] Gomaa A. I., Khan S. A., Toledano M. B., Waked I., Taylor-Robinson S. D. (2008). Hepatocellular carcinoma: epidemiology, risk factors and pathogenesis. *World Journal of Gastroenterology*.

[3] Chen W.-Q., Zeng H.-M., Zheng R.-S., Zhang S.-W., He J. (2012). Cancer incidence and mortality in China, 2007. *Chinese Journal of Cancer Research*.

[4] Zhai X.-F., Chen Z., Li B. (2013). Traditional herbal medicine in preventing recurrence after resection of small hepatocellular carcinoma: a multicenter randomized controlled trial. *Journal of Chinese Integrative Medicine*.

[5] Liu Q., Zhang Y.-B., Ma C.-H., Yue X.-Q., Ling C.-Q. (2005). Analysis of literature on therapeutic methods and medicines of traditional Chinese medicine for primary liver cancer. *Journal of Chinese Integrative Medicine*.

[6] Su S.-B., Jia W., Lu A. P., Li S. (2014). Evidence-based ZHENG: a traditional Chinese medicine syndrome 2013. *Evidence-Based Complementary and Alternative Medicine*.

[7] Chen Z., Wang P. (2012). Clinical distribution and molecular basis of traditional Chinese medicine ZHENG in cancer. *Evidence-based Complementary and Alternative Medicine*.

[8] Ling C. Q., Wang L. N., Wang Y. (2014). The roles of traditional Chinese medicine in gene therapy. *Journal of Integrative Medicine*.

[9] Zhang N. L. (2004). Hierarchical latent class models for cluster analysis. *Journal of Machine Learning Research*.

[10] Xu Z., Zhang N. L., Wang Y. (2013). Statistical validation of traditional chinese medicine syndrome postulates in the context of patients with cardiovascular disease. *The Journal of Alternative and Complementary Medicine*.

[11] Xu Z. X., Liu T. F., Wang Y. Q. (2012). Syndromes classification of TCM inquiry about cardiovascular disease based on the analysis of latent structural model. *Chinese Journal of Information on TCM*.

[12] Wang Y., Zhang N. L., Chen T., Poon L. K. M. (2013). LTC: a latent tree approach to classification. *International Journal of Approximate Reasoning*.

[13] Chen T., Zhang N. L., Liu T. F., Poon L. K. M., Wang Y. (2012). Model-based multidimensional clustering of categorical data. *Artificial Intelligence*.

[14] Chinese Society of Liver Cancer (2001). The standard of clinical diagnosis and staging of primary liver cancer. *Chinese Journal of Hepatology*.

[15] Li D.-T., Ling C.-Q., Zhu D.-Z. (2007). Study on the quantitative evaluation on the degree of TCM basic syndromes often encountered in patients with primary liver cancer. *Chinese Journal of Integrated Traditional and Western Medicine*.

[16] Department of Computer Science and Engineering Lantern 3.1.2 Software (vO.14) [CP/0L]. http://www.cse.ust.hk/~lzhang/tcm/resource.html.

[17] Zhu W. F., Zhang H. M. (2005). The basic features of syndrome factors. *Chinese Journal of Basic Medicine in Traditional Chinese Medicine*.

[18] Zhang N. L., Yuan S., Chen T., Wang Y. (2008). Latent tree models and diagnosis in traditional Chinese medicine. *Artificial Intelligence in Medicine*.

[19] Zhang N. L., Yuan S. H., Wang T. F. (2011). Latent structure analysis and syndrome differentiation for integration of traditional Chinese medicine and western medicine( I): basic principle. *World Science and Technology/Modernization of Traditional Chinese Medicine and Materia Medica*.

[20] Zhang N. L., Xu Z. X., Wang Y. Q. (2012). Latent structure analysis and syndrome differentiation for integration of traditional Chinese medicine and western medicine(II): joint clustering. *World Science and Technology/Modernization of Traditional Chinese Medicine and Materia Medica*.

